# Effect of Exercise on Parkinson’s Disease Tremor: A Meta-analysis Study

**DOI:** 10.5334/tohm.599

**Published:** 2021-04-27

**Authors:** Sajjad Farashi, Leila Kiani, Saeid Bashirian

**Affiliations:** 1 Autism Spectrum Research Center, Hamadan University of Medical Sciences, Hamadan, IR; 2 Dental Implant Research Center, Hamadan University of Medical Sciences, Hamadan, IR; 3 Pejouhesh Institute, Hamadan, IR; 4 Department of Public Health, School of Health Social Determinants of Health Research Center, Hamadan University of Medical Sciences, Hamadan, IR

**Keywords:** Meta-analysis, Parkinson’s disease, Exercise, Tremor

## Abstract

**Background:**

Prior studies suggest that exercise may prevent movement disorders in Parkinson’s disease (PD) patients. In this meta-analysis, the pooled effect of exercise on PD-induced tremor was investigated.

**Method:**

Relevant published studies (n = 7) were retrieved by searching major databases, including Scopus, Web of Science and PubMed from 1985 to November 2020. Studies were eligible if the effect of exercise on PD-induced tremor was studied and sufficient information for calculating the effect size was reported. Furthermore, non-English manuscripts and studies related to non-human species were excluded. The quality of studies was evaluated using the improved Newcastle-Ottawa scale (NOS). In this study, variables such as participant’s age and gender, type of exercise, intervention duration and tremor indices were extracted for each study. Between-study heterogeneity and publication bias were calculated using I^2^ statistic and funnel plot, respectively.

**Results:**

Results showed that hand movement and cycling exercises were effective for reducing tremor amplitude or frequency. When all types of exercises (upper, lower or whole-body movement) were considered, an odds ratio (OR) greater than 1 was obtained (log(OR) = 2, 95% CI: 0.88–3.12), while between-study heterogeneity was high (I^2^ = 78%). By restricting the studies to hand-movement exercises, a lower odds ratio (log(OR) = 1, 95% CI: 0.24–1.77) with small between-study heterogeneity (I^2^ = 0.0%, p = 0.502) was obtained. Statistical analysis based on Egger’s and Begg’s tests revealed no significant publication bias.

**Conclusions:**

Outcomes of this study suggested that exercises as inexpensive, non-invasive and easy-to-implement strategies could be applied for PD patients alongside medical interventions for reducing tremors.

**Clinical Highlights:**

- Exercises reduce tremors in Parkinson’s disease patients significantly.
- Hand movement exercises are beneficial for reducing tremors in PD patients.

## Introduction

Parkinson’s disease (PD) is a neurodegenerative disease that affects movement. One of the main symptoms of PD is tremor that is seen in rest or even during the action [1,2,3]. Tremor reduction has been the subject of several researches. Since pharmacological treatments which use levodopa/carbidopa, dopamine agonists and anticholinergic agents reduce the tremor partially [4], other solutions such as technological-based tremor suppression devices have been proposed that use mechanical damping [5] or electrical stimulation techniques [6] for suppressing tremors in PD patients. Besides the technological advancements, studies have reported the effectiveness of exercises on tremor reduction.

Forced exercises (i.e. aerobic exercises with augmented rate to reach the rate greater than the subject’s voluntary rate) might have promising effects on motor functions of PD patients [7]. Some studies compared the benefits of forced exercises to antiparkinsonian drugs. Using fMRI and brain connectivity, it was revealed that both exercise and medication obtained the same level of motor enhancement and similar changes for connectivity in the brain motor cortex [8]. Furthermore, it was shown that both forced exercise and antiparkinsonian medications trigger similar brain activation, possibly due to the same mechanism of action [9]. Comparison between forced and voluntary exercise revealed that the forced exercise compared with voluntary exercise increased cortical-subcortical activities (motor cortex-thalamus) in PD patients [10]. Furthermore, forced training may enhance inter and intramuscular coordination and in this way improves movement control [11]. Studies in animal models revealed that intensive exercise might attenuate the over-activation of basal ganglia-cortical circuits which could be one of the reasons for tremors in PD patients [12]. Forced exercise possibly enhances the release of neurotrophic factors within the basal ganglia which increases the level of dopamine [13]. Furthermore, forced exercise triggers angiogenesis and synaptogenesis [14], alters neurochemistry and brain connectivity, promotes neuroplastic changes in the brain [8,9,10] and promotes the high-frequency signals from peripheral afferents to the sensorimotor cortex [15].

Some other studies proposed that voluntary exercises might delay the progressive deterioration of the brain in mouse models of PD [16].

In addition, previous studies reported that small but significant volume reduction in the caudate and putamen observed for PD patients [17]. Aerobic exercise and coordination training were proposed as promising tools for increasing basal ganglia volume [18]. Furthermore, aerobic exercise compensates neuromuscular slowing and in this way helps to mitigate movement inabilities in PD patients [19].

Despite the positive effects of exercise on PD tremors that were reported in some studies [7,8,9], some other studies rejected such positive effects [20]. In this study, the effectiveness of exercise on the tremor reduction in PD patients was evaluated using a meta-analysis strategy. To the best of our knowledge, it is the first meta-analysis for investigating the effect of exercise on the tremor of PD patients. Due to the lack of sufficient studies, we did not limit this study to a specific type of exercise and several types of exercises, including cycling, Tango dance, eccentric training, stretching, upper-body karate training and hand movements training during a virtual reality paradigm were included in this meta-analysis. Tremor reduction was evaluated using different measures, including UPDRS scale, hand torque measurement and tremor amplitude and frequency which were obtained by electronic sensors. Results showed positive effects of exercise on tremor reduction.

## Materials and methods

The guidelines for Preferred Reporting Items for Systematic Reviews and Meta-analyses (PRISMA) were used in this meta-analysis [21]. In this study, the relationship between tremor and exercise was evaluated by a random effect model. All measurements and analyses were performed by STATA software version 15 (StataCorp, College Station, TX, USA) considering a significant level of 0.05 (PROSPERO registration number: CRD42021226142).

## Searching Strategy

Different databases including PubMed, Web of Science and Scopus were searched according to the search terms of (tremor) AND (neurodegenerative disease OR Parkinson’s disease OR Parkinsonism OR PD) AND (exercise OR physical activity OR physical therapy).

## Inclusion and exclusion criteria

Only references published in English were considered from 1985 to November 2020. Original research articles, clinical trials and randomized control trials were included in the study. The review papers and meta-analysis papers relevant to PD patients were excluded from this study, while they were carefully checked for suitable and relevant missing references.

## Data collection and validity assessment

The searching procedure was performed by two independent authors (S.F and L.K). Any possible disagreements were resolved by discussion between authors. For papers published between 1985 to November 2020, PICO model (i.e. patients, intervention, comparison and outcome model) was used. In the search strategy, participants were PD patients, the intervention was exercise (forced, voluntary or aerobic), measures were tremor amplitude or frequency or Unified Parkinson’s Disease Rating Scale (UPDRS) and the outcome was tremor change after intervention. Due to the lack of sufficient studies, both Randomized Controlled Trials and observational studies were included in this meta-analysis. For each eligible study, information regarding first author, publication year and study sample size, participant information including age and gender, odds ratio (OR) and 95% confidence interval (CI) were extracted using an extraction form. For assessing the quality of each study, the improved Newcastle-Ottawa Statement Manual scale (NOS) was used [22]. According to such scale, election, comparability and outcomes were considered and the score for each study was calculated. The score can be from 0 to 9 while high-quality sources are highlighted by scores ≥7.

## Heterogeneity and publication bias

The heterogeneity between studies was evaluated by Q-test and I^2^ statistic [23] in a way that for I^2^ values of 25%, 50% and 75%, heterogeneity was considered as low, moderate and high, respectively. Furthermore, the assessment of publication bias was performed by funnel plot, as well as Begg’s and Egger’s tests. Begg’s test uses the rank correlation of log(OR) and its variance, while Egger’s test uses the linear regression of log(OR) with a weighted standard error of log(OR) [24].

## Statistical analysis

Considering the eligible studies, the pooled OR (log(OR)) and 95% CI were obtained using the random effect model. The effect size (*d*) was calculated using Cohen’s d formula which considers the mean difference between groups divided by the pooled standard deviation (Eq 1).

1 d = (\overline {{X_1}} - \overline {{X_2}})/Pooled\ SD

Where

2 Pooled\ SD = \sqrt {\frac{{\left({{n_1} - 1} \right)S{D_1}^2 + \left({{n_2} - 1} \right){SD_2}^2}}{{{n_1} + {n_1} - 2}}}

In (1) and (2), {\bar X_t}, *SD_t_* and *n_t_* refer to mean tremor measure, standard deviation of tremor measure and sample size for group *t*, respectively. The effect size was calculated for finding the effect of exercise intervention on tremors between exposed and control PD groups (independent samples) or for pre-exposed and post-exposed PD patients (dependent samples). The 95% CI was also calculated according to the Hedge and Olkin formula as follows [25].

3 \sigma \left(d \right) = \sqrt {\frac{{{n_1} + {n_2}}}{{{n_1} \times {n_2}}} + \frac{{{d^2}}}{{2\left({{n_1} + {n_2}} \right)}}}

95\% CI:d \pm 1.96\sigma \left(d \right)

The OR was obtained from effect size by the proposed modified method by Hasselblad and Hedges [26,27] under the assumption of logistic (near normal) distribution and equal variances for both groups using the following formulas.

4 \log \left({OR} \right) = d\pi /\sqrt 3

5 V\left({\log \left({OR} \right)} \right) = {V_d}{\pi ^2}/3

In which *d* is the standard effect size, *V_d_* is the variance of the standard effect size and π is a constant (~3.14).

## Results

**Figure 1:**
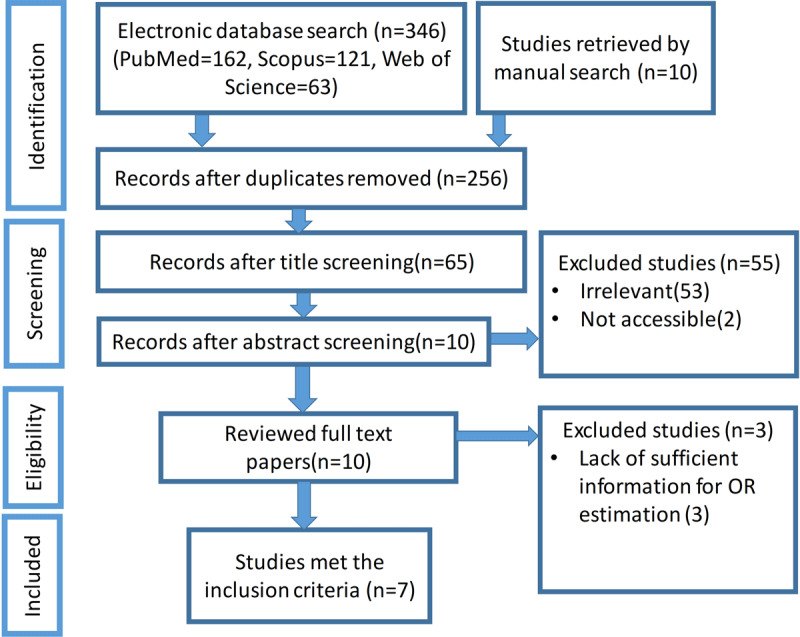
Flow diagram for different phases of the performed systematic review.

In the present study, the initial search for investigating the effect of exercise on tremors obtained 346 records. After excluding duplicate articles and not relevant papers from title, abstract or full-text screening, seven articles were found as eligible studies for further analyses (see ***Figure 1***). Among seven remained manuscripts, 3 were randomized control trials (RCT), and four were case-control (CC) studies (***Table 1***). The total sample size of this study was 111 PD people. The summary of eligible studies was reported in ***Table 1***. It should be noted that among seven remained studies, two of them (i.e. Palmer et al. and Cikajlo et al.) studied the effect of two types of exercises on PD tremor; therefore, overall, nine values for the effect of exercise on tremor reduction were retrieved for this meta-analysis. A brief summary of included studies is as follows.

**Table 1:** Summary of studies.


Author, Year, Ref	Design	Sample Size (gender, m/f)	Age	Type of exercise	Intervention duration	Types of tremor	Quality	Measure	Main outcomes

Palmer, 1986, [28]	CC	7 (6, 1)	63.9 ± 4.4, 65.9 ± 7.2	slow stretching or upper body karate training in a seated situation	15-min warm-up, 35-min of karate training and 10-min of cool-down stretching exercise	Resting tremor	High	Rectified envelope of alternating torque while the patient is evaluated by a mental activation task.	Both types of exercises were effective for tremor reduction.

King, 2009, [29]	CC	20 (14, 6)	64.65 ± 8.56	Vibration by sound waves	5 one-minute	Resting, action, and posture tremor	High	UPDRS tremor score	Improvements were seen in motor control symptoms at the time of assessment.

Duncan, 2012, [30]	RCT	26(15,11)	69.3 ± 1.9	Tango exercise	1-hour, twice weekly for 12 months	Resting, action, and posture tremors	High	MDS-UPDRS-3 items 3.15-3.18	Tremor scores decreased slightly following performing Tango.

Ridgel, 2012, [15]	CC	10(4, 6)	64 ± 2.1	Cycling exercise	30-min pedaling exercise followed by 5-min warm up/cool down session	Resting tremor	Low	Accelerometer and gyroscope sensors	Immediate improvement in tremor and bradykinesia was reported after a single bout of cycling.

Stuckenschneider, 2015, [20]	CC	10(4, 6)	71.3 ± 4.93	movements similar to cycling	12-week active or passive forced exercise, 40-min, three times per week	Resting and postural tremors	High	Frequency of tremor using electronic instrument	Only kinetic tremor was reduced significantly after exercise while rest and postural tremors were not changed significantly.

Cikajlo, 2019, [31]	RCT	10(5, 5)	69.3 ± 1.9	Hand movements using a virtual cube pick and place task	10-section, 3 weeks training strategy using 3D Oculus Rift glass (group1) or 2D laptop screen (group2)	Resting tremor	Low	UPDRS motor section and motion trajectory analysis	2D environment reduced average tremor better than the 3D environment.

Kadkhodaie, 2020, [32]	RCT	11 (4, 7)	67.82 ± 9.64	limb pure eccentric training	33-45 minutes sessions for a 6-week period, 3 days per week	Resting and postural tremors	High	Amplitude obtained by accelerometer	After the exercise, a significant reduction in resting tremor amplitude was obtained in the intervention group.


Abbreviations: Randomized control trial (RCT), Case-control (CC), Unified Parkinson’s disease rating scale (UPDRS), Movement Disorder Society-Unified Parkinson’s Disease Rating Scale Item 3(MDS-UPDRS-3), 3 dimensional (3D), two dimensional (2D), male/female (m/f).

Palmer et al. used hand movement training in karate and stretching exercises (15-minute warm-up stretching exercise, 35-minute karate exercise and 10-minute cool-down exercise for 6 to 12 weeks) and reported that even in a sitting position, exercise could reduce the resting tremor of individuals with PD [28]. In the study performed by King et al. [29], vibroacoustic therapy was used in 5 one-minute sessions. Improvements were observed in the motor control symptoms following the intervention. Duncan et al. [30] used Tango exercise in 1-hour sessions, twice weekly for a 12-month period and showed that tremors reduced after the intervention. Ridgel et al. showed the positive effect of active-assisted cycling exercise on the reduction of resting tremors [15]. They used a motorized bike and forty minutes of forced intervention. Stuckenschneider et al. used active forced exercise for 12 weeks (40-minute, three times per week) and reported that forced exercise based on the increased frequency of cadence had a positive effect on kinetic tremor in PD patients [20]. Cikajlo et al. used a pick and place exercise in a virtual reality paradigm (10 sessions, 3 weeks) [31] and Kadkhodaie et al. used eccentric training exercise (for 3 days/week for 6 weeks, 35 to 45 minutes per session) [32]. These studies showed that exercises based on hand actuation were suitable strategies for reducing resting tremors in PD patients [31,32].

**Figure 2:**
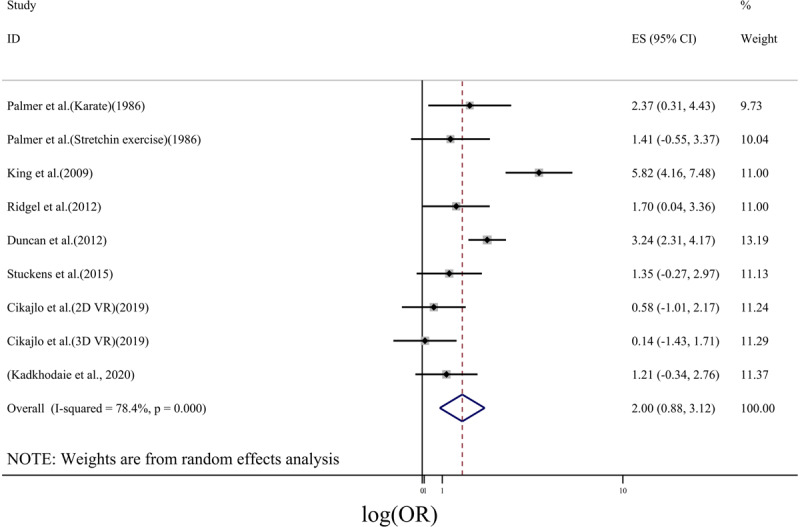
Forest plot for the effect of exercise on tremor. The horizontal axis is the crude odds ratio.

**Figure 3:**
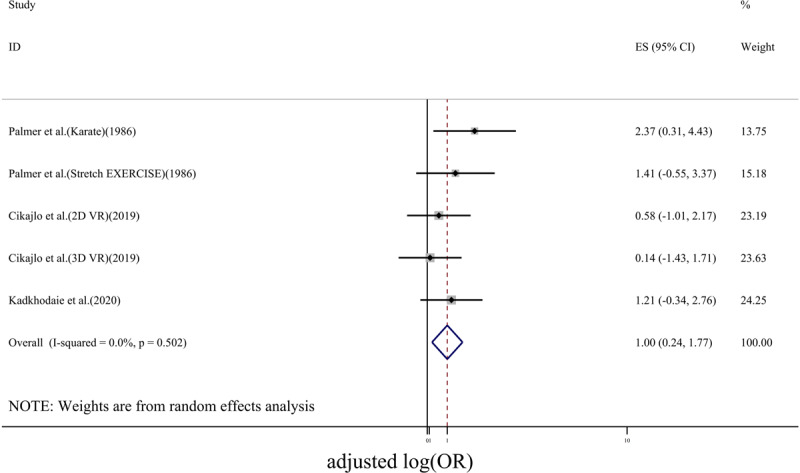
Forest plot for the effect of hand movement-based exercise on tremor. The horizontal axis is the adjusted odds ratio.

The efficacy of exercise intervention for tremor reduction was evaluated based on log(OR). In ***Figure 2***, the forest plot for the effect of exercise on tremor was depicted. Using a random effect model, a pooled log(OR) of 2 (95% CI: 0.88–3.12) was obtained. However, the heterogeneity of studies was relatively high (I^2^ = 78.4%, p < 0.01). Studies included in this meta-analysis used different types of exercises including whole-body exercise (Tango dancing [30]), lower body activity via cycling [15,20], exercises focused on hand movements [28,31,32] and whole-body activity induced by acoustic vibration [29]. In order to investigate the source of between-study heterogeneity, only studies in which exercises were done based on hand movements were included into the subgroup analysis. For this case, the forest plot was depicted in ***Figure 3***. For this subgroup analysis, the heterogeneity was reduced to a low value (I^2^ = 0.0%, p = 0.502), while log(OR) was 1.00(95% CI: 0.24–1.77).

**Figure 4:**
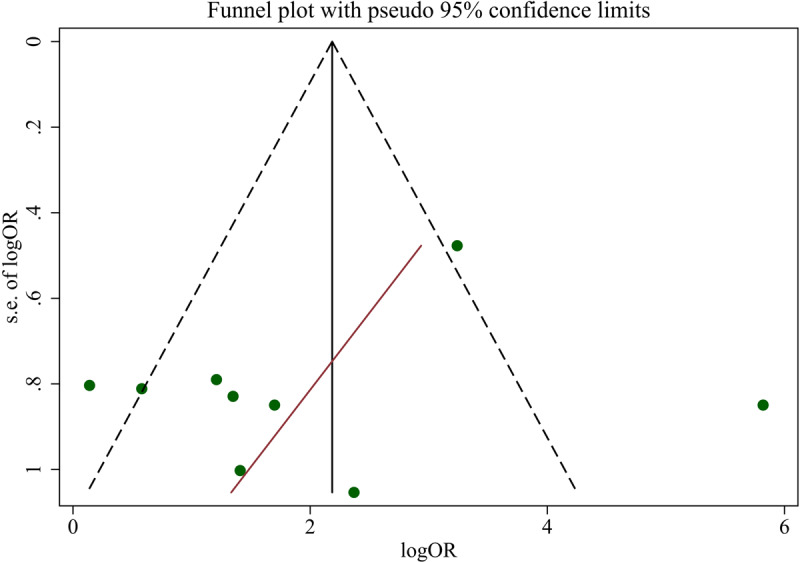
Funnel plot for the effect of exercise on tremor for PD patients. The line inside the funnel plot shows the regression line.

The publication bias assessment was performed using the funnel plot (see ***Figure 4***) and Begg’s and Egger’s tests. When all studies were considered, no publication bias was found by Egger’s and Begg’s test (p-value: 0.386 and 0.295, respectively). Also, for subgroup analysis, these tests detected no publication bias (p-value: 0.11 and 0.221, respectively).

## Discussion

According to this meta-analysis, exercises had positive, statistically significant effects on tremor reduction in patients with PD. When all types of movements including upper body and lower body activities (i.e. hand movement and cycling) and the whole-body activity (i.e. dance or whole-body exposed to vibroacoustic stimulation) were included, the log(OR) of 2 (95% CI: 0.88–3.12) was obtained while the heterogeneity of studies in such a case was relatively high (I^2^ = 78%). When more relevant studies i.e. studies that used only hand movement-based exercises were considered, log(OR) of 1 (95% CI: 0.24–1.77) was obtained with relatively low between-study heterogeneity (I^2^ = 0.0%, p = 0.502). The publication bias assessment using the funnel plot and Begg’s and Egger’s test showed that there was no publication bias.

Considering all studies, even though a larger effect was obtained, the confidence interval for log(OR) was larger than the one that was obtained for studies containing only hand movement-based exercises. Larger confidence interval indicated a lower level of precision of log(OR) [33] when all studies were used for meta-analysis. From ***Figure 2***, the largest effect of exercise on tremor reduction was reported by King et al. (2009) as it was 5.82(95% CI: 4.16–7.48). In subgroup analysis, the study of King et al was not considered since the type of intervention was not relevant to the hand movement-based exercises. This might be the reason that why pooled effect size obtained by subgroup analysis was reduced compared with the analysis when all studies were included.

PD patients have many problems with visual tasks such as navigating [34]. Exercises such as hand movement for grasping and picking up an object employ several circuits for performing such visuomotor transformation. These circuits consist of the parietofrontal circuit, ventral premotor cortex (F5 area), object recognition system (ventral occipito-temporal), the system for goal-directed control (dorsal occipito-parietal region) [35] and putamen, ventral thalamus [36] and many others. Therefore, it was not surprising that a hand movement exercise for grasping and picking up an object (i.e. the work of Cikajlo et al.) [31] showed a positive effect on movement disorders in PD patients, possibly by affecting the thalamus and basal ganglia subdomains. Furthermore, the cycling forced exercises (i.e. the work of Ridgel et al [37]. and Stuckenschneider et al. [20]) may induce cortical and subcortical activation and in this way improves motor functions in PD patients [38].

In addition to positive effects of exercise on tremors, evidences showed positive effects of exercise on other symptoms of PD. Palmer et al. [28] reported the improvement of gait ability, motor coordination and grasp strength for activities required fine control. Hand movement-based exercises combined with cognitive load (as was the case of Cikajlo et al [31]. in grasping and picking up exercise) showed positive effects on UPDRS scores for upper limb [31]. Exercises based on eccentric rehabilitation, as was the case of Kadkhodaie et al. [32], showed a positive effect on hand tremor amplitude, however, no statistically significant effect was observed for postural tremor. The active-assisted cycling exercise (i.e. the study of Ridgel et al. [15])showed enhancement for both tremor and bradykinesia, while the result of another study showed that gait velocity and stride length were also enhanced following the forced exercise [20]. In addition, dance exercise like Tango that incorporated both physical activities and cognitive challenges showed enhanced balance, gait, bradykinesia and also rigidity [30].

Despite the overall positive effect of exercise on tremor reduction in PD patients that was obtained by this meta-analysis, the heterogeneity between studies considering design, intervention duration and measures were relatively high. This limits a final conclusion for proposing the best type of exercise or the optimal intervention for a tremor reduction strategy.

Furthermore, according to the systematic search, limited numbers of eligible studies with a total small population sample size were retrieved. The bigger sample size that needs more researches in the future may increase our understanding of the way that exercise influences tremor reduction.

## Conclusion

Current meta-analysis showed that exercise might have positive effects on tremor reduction in patients with PD. However, bigger sample size is needed to interpret the possible mechanisms following each type of exercise (i.e. aerobic vs. anaerobic, forced vs. voluntary, active vs. passive). Furthermore, different measures that were used for tremor assessment (i.e. tremor amplitude, tremor frequency, or UPDRS score) might bias the obtained result of this study.
